# In Vitro Lipid Overload Affects Cellular Proliferation, Apoptosis, and Senescence in a Time-Dependent Manner in HepG2 Hepatocytes and LX-2 Hepatic Stellate Cells

**DOI:** 10.3390/cells13030282

**Published:** 2024-02-04

**Authors:** Adriana Campos-Espinosa, Carolina Guzmán, Karla Zaira Medina-Ávila, Gabriela Gutierrez-Reyes

**Affiliations:** Laboratorio de Hígado, Páncreas y Motilidad, Unidad de Medicina Experimental, Facultad de Medicina, UNAM, Hospital General de México “Dr. Eduardo Liceaga”, Ciudad de México 06720, Mexico; adria.camp88@hotmail.com (A.C.-E.); carova@prodigy.net.mx (C.G.); kzairamedina@gmail.com (K.Z.M.-Á.)

**Keywords:** steatosis, hepatocytes, hepatic stellate cells, apoptosis, proliferation, senescence

## Abstract

Different cellular mechanisms influence steatotic liver disease (SLD) progression. The influence of different levels of steatogenic inputs has not been studied in hepatocytes and hepatic stellate cells (HSCs). Methods: HepG2 hepatocytes and LX-2 HSCs were cultured in mild (MS) and severe (SS) steatogenic conditions. TGF-β stimulation was also tested for HSCs in control (T) and steatogenic conditions (MS-T and SS-T). Steatosis was stained with Oil Red, and the proliferation was assayed via WST-8 reduction, apoptosis via flow cytometry, and senescence via SA-β-galactosidase activity. Results: Regarding hepatocytes, steatosis progressively increased; proliferation was lower in MS and SS; and the viability of both conditions significantly decreased at 72 h. Apoptosis increased in MS at 72 h, while it decreased in SS. Senescence increased in MS and diminished in SS. Regarding HSCs, the SS and SS-T groups showed no proliferation, and the viability was reduced in MS at 72 h and in SS and SS-T. The LX-2 cells showed increased apoptosis in SS and SS-T at 24 h, and in MS and MS-T at 72 h. Senescence decreased in MS, SS, and SS-T. Conclusions: Lipid overload induces differential effects depending on the cell type, the steatogenic input level, and the exposure time. Hepatocytes are resilient to mild steatosis but susceptible to high lipotoxicity. HSCs are sensitive to lipid overload, undergoing apoptosis and lowering senescence and proliferation. Collectively, these data may help explain the development of steatosis and fibrosis in SLD.

## 1. Introduction

Metabolic-dysfunction-associated steatotic liver disease (MASLD), formerly known as nonalcoholic fatty liver disease, is the most common hepatic affection worldwide [1,2,3]. This disease is characterized by a succession of events starting with hepatic lipid accumulation, which affects more than 5% of hepatocytes, known as simple steatosis [4]. Additional inflammation and hepatocyte ballooning is observed in the steatohepatitis stage, which can be accompanied by fibrosis and followed by cirrhosis, hepatocellular carcinoma, and end-stage liver failure [4,5]. Liver steatosis is the key event defining the course of the disease [5]. Lipid accumulation depends on different metabolic inputs, including insulin resistance, dyslipidemia, and obesity [6,7,8,9]. Under these conditions, the bioavailability of dietary free fatty acids (FFAs) is increased through adipose tissue lipolysis or synthesis via hepatic de novo lipogenesis [7,9,10]. Hepatocytes and every other hepatic cell, including hepatic stellate cells (HSCs), are exposed to the steatogenic environment. Hepatocytes and HSCs have distinct roles in the pathogenesis of chronic liver disease. In the steatotic liver, hepatocytes assume the majority of the metabolic challenge that excessive lipid levels represent for the liver. However, when its metabolic capacities are exceeded, the accumulation of lipid droplets occurs [7]. Quiescent HSCs are considered a retinoid depot in a healthy liver, while in chronic liver disease, including MASLD, HSCs are activated mainly by transforming growth factor (TGF)-β, becoming the major source of extracellular matrix proteins leading the fibrogenic response [11]. Different cellular mechanisms have been described in the progression of chronic liver diseases, including two types of cell death, apoptosis and necrosis [10,12], and senescence [13,14]. However, the cellular outcomes of the exposure of these cell types to different steatogenic environments have not been fully described.

In this study, we investigated the cellular effects of two levels of steatogenic conditions—mild and severe—on different processes involved in the development of chronic liver diseases—proliferation, apoptosis, and senescence—in hepatocytes and HSCs in vitro. This model showed lipid accumulation in HepG2 hepatocytes of >5% in mild conditions and up to 40% in severe conditions [15].

## 2. Materials and Methods

### 2.1. Cell Culture

Human hepatoma HepG2 cells were obtained from ATCC (HB-8065, ATCC, Manassas, VA, USA). HepG2 cells were cultured in RPMI 1640 (Gibco, San Diego, CA, USA) supplemented with 10% fetal bovine serum (FBS, Gibco, CA, USA) and 1% antibiotics (penicillin–streptomycin, 10,000 U/mL; Gibco, CA, USA). LX-2 HSCs (kindly donated by Dr. Scott Friedman) were cultured in Dulbecco’s Modified Eagle’s Medium (DMEM, Gibco, CA, USA), supplemented with 2% FBS and 1% antibiotics. Two lipid mixes were prepared to further supplement either the RPMI or DMEM previously prepared, rendering two levels of steatogenic inputs: mild steatosis (MS) and severe steatosis (SS), as described in [15]. The lipid mix contained sodium oleate (Santa Cruz Biotechnology, Santa Cruz, CA, USA) and sodium palmitate (Santa Cruz Biotechnology, Santa Cruz, CA, USA) at a 2:1 ratio. The MS mix contained 50 µmol of the lipid mix per liter of medium, whereas the SS mix’s final concentration was 500 µM.

Cells were cultured under standard conditions at 37 °C and 5% CO_2_. Before steatogenic exposure, the cells were seeded and cultured for 24 h in control conditions to ensure the adhesion of the cell to the dish. The medium was then replaced with the steatogenic enriched version. Cells were incubated for a further 24, 48, and 72 h. In all cases, the medium was replaced with fresh medium every 24 h to ensure lipid availability and accumulation.

Three groups were established for HepG2 cells: the control (C), cultured in RPMI without lipids; MS, containing RPMI + the MS lipid mix; and SS, containing RPMI + the SS lipid mix. For LX-2 HSCs, a control stimulated with 2.5 ng/mL of TGF-β (Peprotech, Cranbury, NJ, USA) was included for each condition and time, rendering the following wells: C, the control, cultured in DMEM without lipids; T, containing DMEM without lipids + TGF-β; MS, containing DMEM + the MS lipid mix; MS-T, containing DMEM + the MS lipid mix + TGF-β; SS, containing DMEM + the SS lipid mix; and SS-T, containing DMEM + the SS lipid mix + TGF-β. All experiments were performed in triplicate in 2–3 independent assays.

### 2.2. Lipid Contents

To assess lipid accumulation, cells were cultured on coverslips in 24-well plates. The cells were stained with Oil Red (Abcam, Waltham, MA, USA). The cells were fixed with 4% paraformaldehyde for 1 h, incubated with 70% isopropanol for 5 min, and stained with Oil Red for 30 min. A morphometric analysis was performed to quantify the amount of stained lipids. Ten optic fields were digitalized from every well. The images were analyzed using ImageJ, v1.53c, and the percentage of the area stained red was assessed.

### 2.3. Cellular Growth and Viability

The percentage cellular viability/mortality was initially assessed via staining with Trypan blue 0.4%. Cell proliferation was analyzed via the reduction in tetrazolium salt WST-8 using the Cell Counting Kit (CCK, Sigma-Aldrich, St. Louis, MO, USA) according to the manufacturer’s instructions. A total of 1000 cells for HepG2 or 5000 for LX-2 were seeded per well in 96-well plates and cultured in the different conditions for 24, 48, and 72 h. An aliquot of 10 µL of CCK was added per well, and the wells were incubated for 4 h at 37 °C and 5% CO_2_. The absorbance was read at 450 nm with a microplate reader. A seven-point standard curve (1000–24,000 cells) was used to interpolate the data and calculate the cell number. 

### 2.4. Cell Death

Apoptosis and necrosis were assessed with an Apotracker Green Kit and 7-AAD (Biolegend, San Diego, CA, USA). Cells collected from the different conditions were washed in phosphate-buffered saline (PBS) and resuspended in 50 µL of staining buffer. An aliquot of 5 µL of Apo15 was added, and the mixture was incubated for 20 min at room temperature while protected from light. A wash with PBS was followed by resuspension in 300 µL of staining buffer with 5 µL of 7-AAD. The samples were read with a FACS Canto II (BD, USA) and analyzed using FACSDiva v.6.0 software. A total of 20 × 10^3^ events were acquired in triplicate from 3 × 10^5^ cells. Apo15-positive staining indicated the presence of phosphatidylserine in the outer plasma membrane. Apo15+/7AAD- corresponded to early apoptosis, whereas Apo15+/7AAD+ corresponded to late apoptosis and Apo15-/7AAD+ to necrosis.

### 2.5. Cellular Senescence

Senescence-associated beta-galactosidase (SA-βgal) was detected with the Senescence Detection Kit (Abcam, MA, USA) in cells cultured on coverslips for every treatment and exposure time. The cells were fixed with 500 µL of a fixative solution for 15 min at room temperature. The cells were washed twice in 1 mL of 1X PBS, and 500 µL of the staining solution mix was added to each well. The plates were sealed and introduced into a hermetic bag, extracting all air to avoid the effect of atmospheric CO_2_ on the pH inside the wells. The plates were incubated overnight at 37 °C. A blue color developed in senescent cells, and counterstaining was performed with hematoxylin. A morphometric assessment was performed in 10 digitalized optic fields per well and analyzed using ImageJ v.1.53c.

### 2.6. Statistics

Data were presented as means ± standard deviation. For the morphometric analysis performed for steatosis and senescence, the averaged percentage of the area corresponding to positive staining was calculated from 10 optic fields per well. The Shapiro–Wilk test was performed to assess normality. Normally distributed data were analyzed using one-way ANOVA, followed by Tukey’s post hoc test. The Kruskal–Wallis test, followed by Dunn’s post hoc test, was applied for non-normally distributed data. Statistical analyses were performed using GraphPad Prism v.9.5.1. *p* < 0.05 was considered significant.

## 3. Results

### 3.1. Steatosis in HepG2 Cells

**Figure 1:**
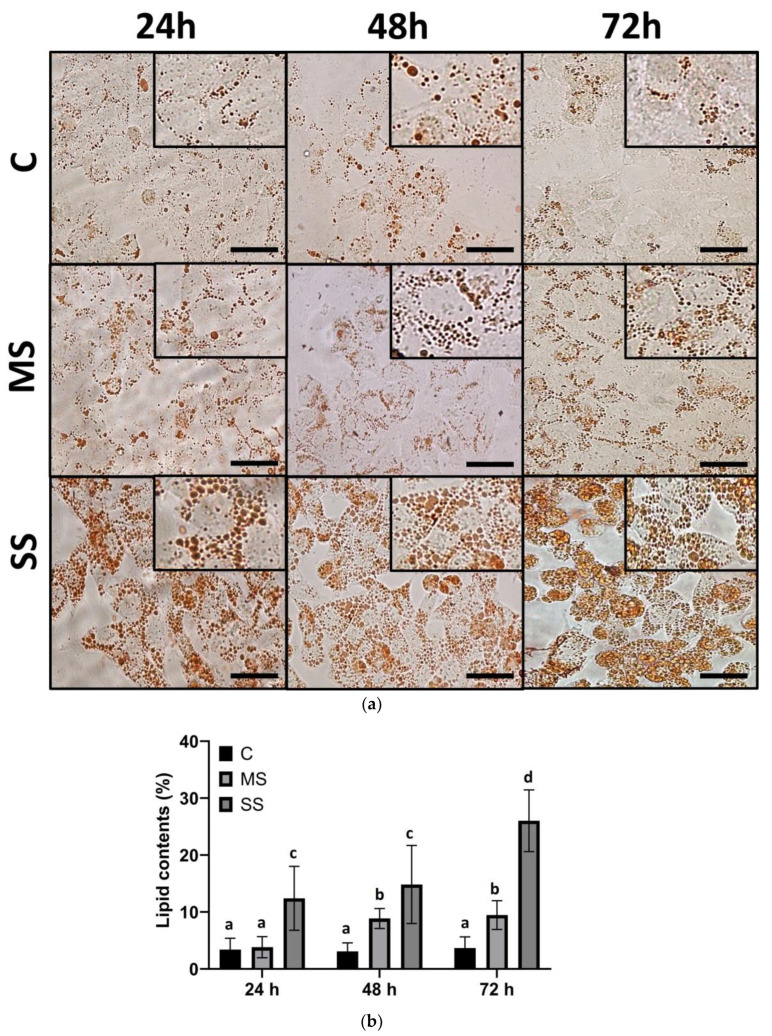
Steatosis. HepG2 cells were cultured in control and steatogenic conditions: mild steatosis (MS: 50 µM of 2:1 mix of oleate–palmitate) or severe steatosis (SS: 500 µM of 2:1 mix of oleate–palmitate). A morphometric assessment was conducted on slides stained with Oil Red. (**a**) Representative micrographs of steatosis levels in HepG2 cells. (**b**) Lipid contents. Data are shown as means ± SD and were analyzed using one-way ANOVA followed by Tukey’s post hoc test. Bars not sharing a letter are significantly different; *p* < 0.05. *n* = 9 wells from three independent assays in triplicate per condition and culture time. Bar = 50 µm.

The HepG2 cells cultured in steatogenic conditions showed a significant increase in intracellular lipid contents (Figure 1a). MS induced a two-fold increase in intracellular fat at both 48 and 72 h compared with the control condition. SS induced a three-fold increase in fat content at 24 and 48 h and an almost five-fold increase at 72 h compared with the control (Figure 1b). The LX-2 cells incubated in steatogenic conditions did not show lipid staining with Oil Red.

### 3.2. Proliferation and Viability

**Figure 2:**
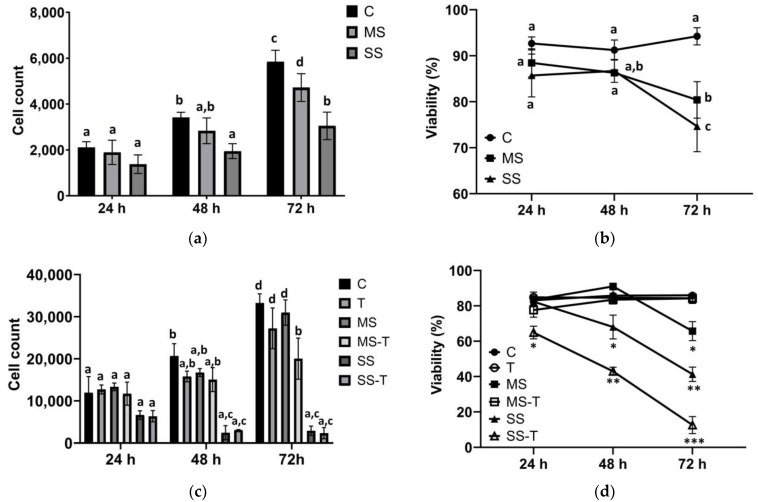
Proliferation and viability. HepG2 cells were cultured in control (C) and steatogenic conditions: mild steatosis (MS) or severe steatosis (SS). LX-2 cells were cultured in C, MS, and SS conditions. The addition of TGF-β rendered 3 further groups: T, MS-T, and SS-T. Cellular proliferation was assayed, and viability was assessed in both HepG2 and LX-2 cells. (**a**) Proliferation of HepG2 cells. (**b**) HepG2 viability. (**c**) Proliferation of LX-2 cells. (**d**). LX-2 viability. Data are shown as means ± SD and were analyzed using one-way ANOVA followed by Tukey’s post hoc test. Bars not sharing a letter are significantly different; *p* < 0.05. * *p* < 0.05 vs. C, T, and MS-T; ** *p* < 0.05 vs. SS at 24 and 48 h; and *** *p* < 0.05 vs. SS-T at 24 and 48 h. *n* = 6 wells from two independent experiments.

HepG2 cells showed increased proliferation during the study period, regardless of the culture conditions. However, the proliferation rate decreased by 24% in MS and 60% in SS (Figure 2a). Accordingly, viability was decreased in HepG2 cells exposed to MS and SS at 72 h compared with C (*p* < 0.0001) and the same lipid dose at 24 h (*p* < 0.05; Figure 2b).

LX-2 HSCs were more sensitive to the steatogenic conditions compared with hepatocytes. TGF-β-induced activation of LX-2 was identified as an increased expression of α-smooth muscle actin and was observed in both T and SS-T conditions compared with their respective controls (Figure S1). Control LX-2 cells showed a significant and progressive increase in cell counts from 24 to 72 h. Similar results were observed in T and MS (Figure 2c). However, MS-T showed a 52.9% diminished rate of proliferation compared with MS, being significantly low at 72 h. SS and SS-T did not show proliferation (Figure 2c). T did not modify the viability of LX-2 cells. A similar result was observed in MS-T. However, MS showed lower viability, reaching 40% of live cells at 72 h, compared with MS-T. The MS results suggest that despite the lower proliferation rate, the viability was improved by TGF-β, which did not occur in its absence. Regarding SS, the viability was drastically affected by severe lipid overload. At 24 h, the viability of SS was comparable to that of the control and MS. However, a steady decrease during incubation was observed. TGF-β stimulation in severe steatogenic conditions diminished the cell viability to 61.95 ± 6.52% at 24 h and 6.25 ± 7.98% at 72 h, suggesting that TGF-β is not sufficient to overcome lipotoxicity in severe conditions, as observed in mild conditions (Figure 2d).

### 3.3. Apoptosis

**Figure 3:**
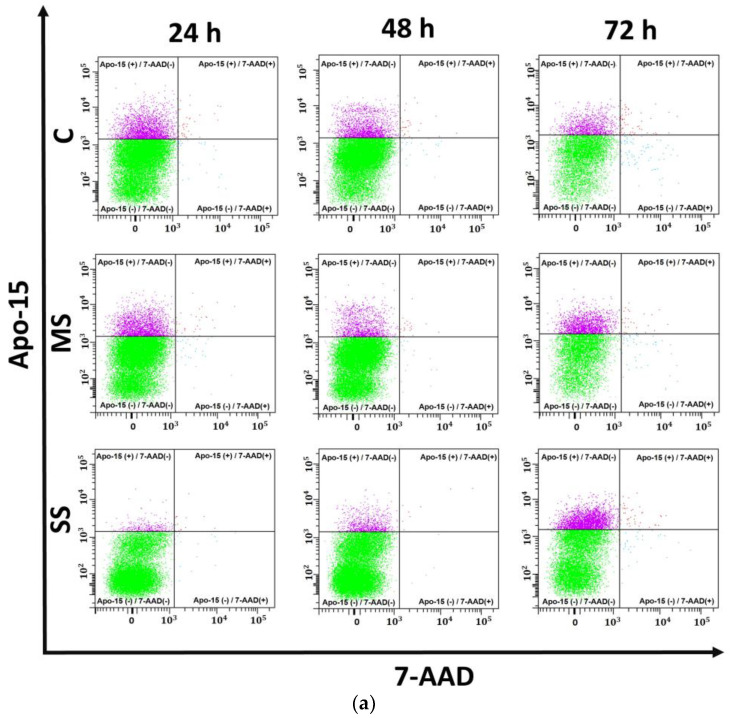

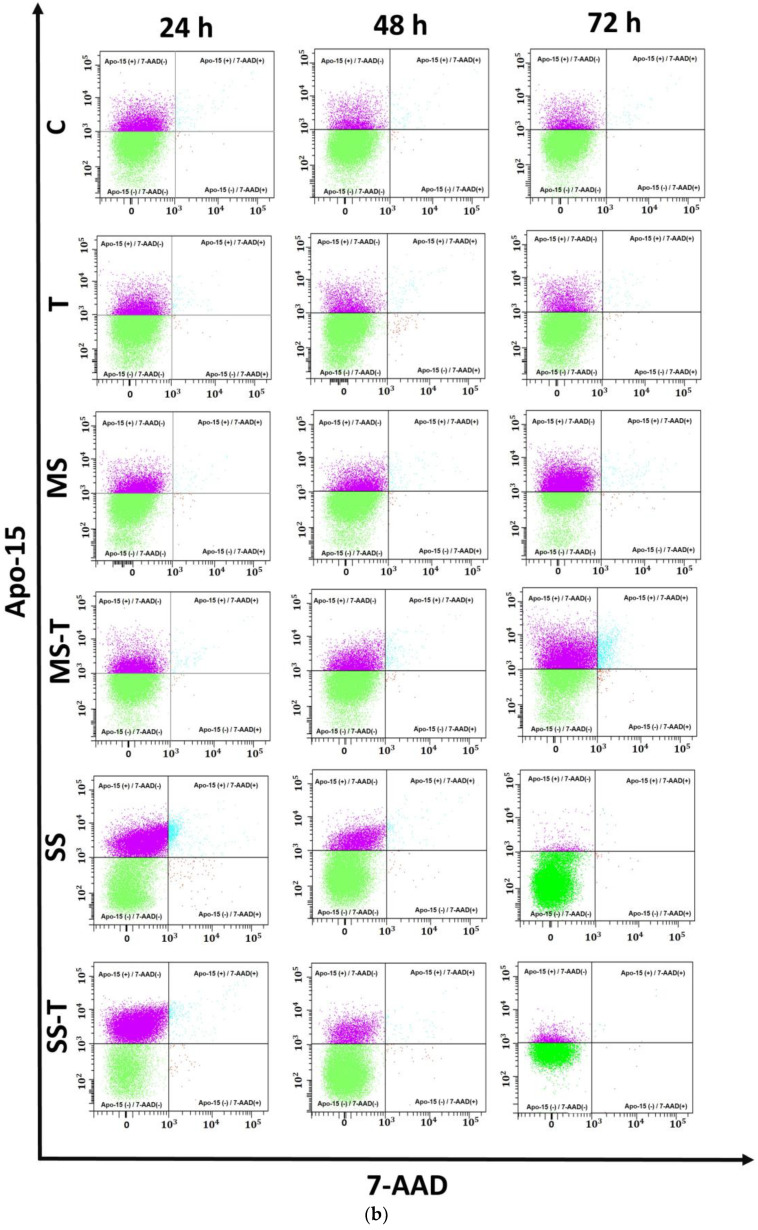

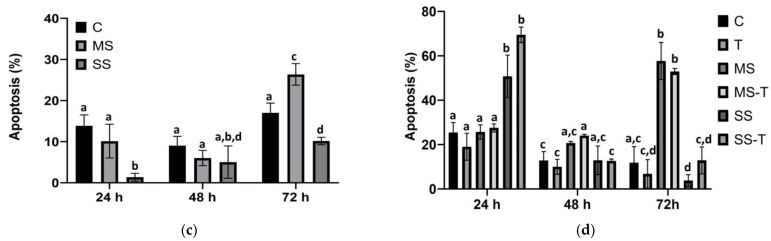
Apoptosis. HepG2 cells were cultured in control (C), mild steatosis (MS), or severe steatosis (SS) conditions. LX-2 cells were cultured in C, MS, and SS conditions. The addition of TGF-β rendered 3 further groups: T, MS-T, and SS-T. Apoptosis was assayed using flow cytometry as the percentage of cells that were phosphatidylserine-positive (Apo15+) and -negative in 7AAD staining (7AAD-). (**a**) Dot plots of HepG2 cells. (**b**) Dot plots of LX-2 cells for different conditions and incubation times. In every dot plot: lower left quadrant—viable cells (Apo15-/7AAD-); upper left quadrant—early apoptosis (Apo15+/7AAD-); upper right quadrant—late apoptosis (Apo15+/7AAD+); and lower right quadrant—necrosis (Apo15-/7AAD+). (**c**) Percentage of apoptosis in HepG2 cells. (**d**) Percentage of apoptosis in LX-2 cells. Data are shown as means ± SD and were analyzed using one-way ANOVA followed by Tukey’s post hoc test. Bars not sharing a letter are significantly different; *p* < 0.05. *n* = 6 wells from two independent experiments.

HepG2 cells showed increased cell death, identified as early apoptosis, in the MS group at 72 h compared with C and MS at 24 and 48 h. SS early apoptosis was significantly lower at 24 h compared with C and MS. Although the Apo15+/7AAD- HepG2 cells had increased by 72 h in SS compared with SS at 24 h, the percentage early apoptosis remained significantly lower compared with those of C and MS (Figure 3a,c). Thus, mild steatosis increases apoptosis, whereas severe steatosis reduces this type of cell death, suggesting that severe steatosis might induce a different type of cell death. No differences in late apoptosis (Apo15+/7AAD+ cells) or necrosis (Apo15-/7AAD+ cells) were observed in hepatocytes (Figure 3a).

Regarding LX-2 cells, apoptosis decreased slightly with the incubation time in C and T cells. Exposure to steatogenic conditions produced differential effects related to both the time and severity of the steatogenic input. MS and MS-T significantly increased apoptosis, showing 57.67 ± 8.35% apoptotic cells in MS and 52.93 ± 1.37 in MS-T at 72 h, whereas SS and SS-T increased apoptosis at 24 h, showing 50.77 ± 9.52% in SS and 69.43 ± 3.50 in SS-T (Figure 3b,d). This is a plausible explanation for the low proliferation and viability observed in SS and SS-T. The percentage of late apoptosis (Apo15+/7AAD+) decreased in SS versus MS at 72 h (MS = 0.50 ± 0.10 and SS = 0.08 ± 0.03; *p* < 0.01; Figure 3b). No differences in necrosis (Apo15-/7AAD+) were observed in LX-2 cells (Figure 3b).

### 3.4. Senescence

**Figure 4:**
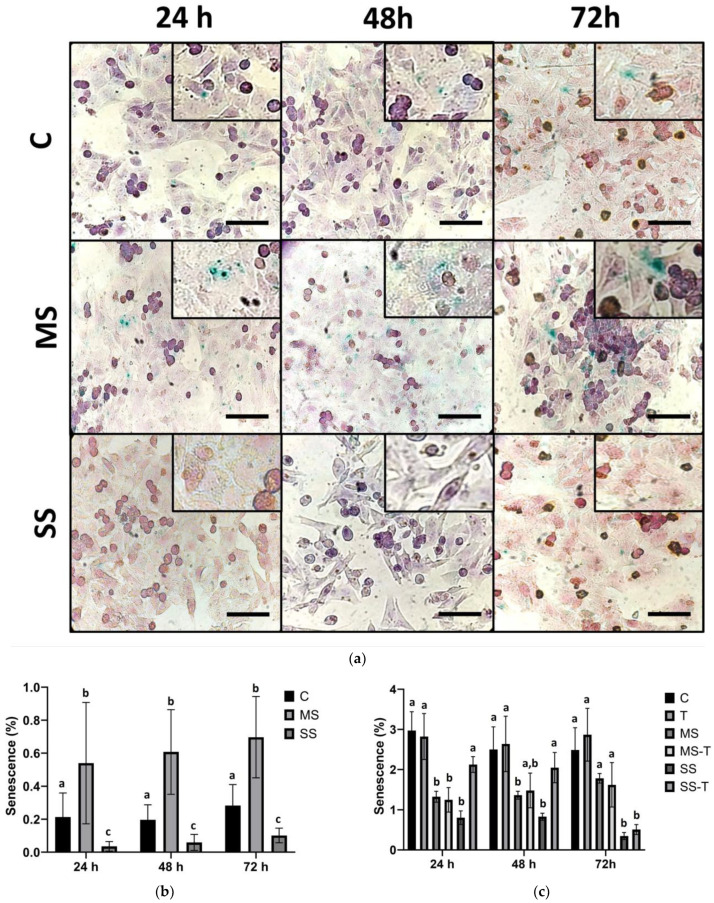

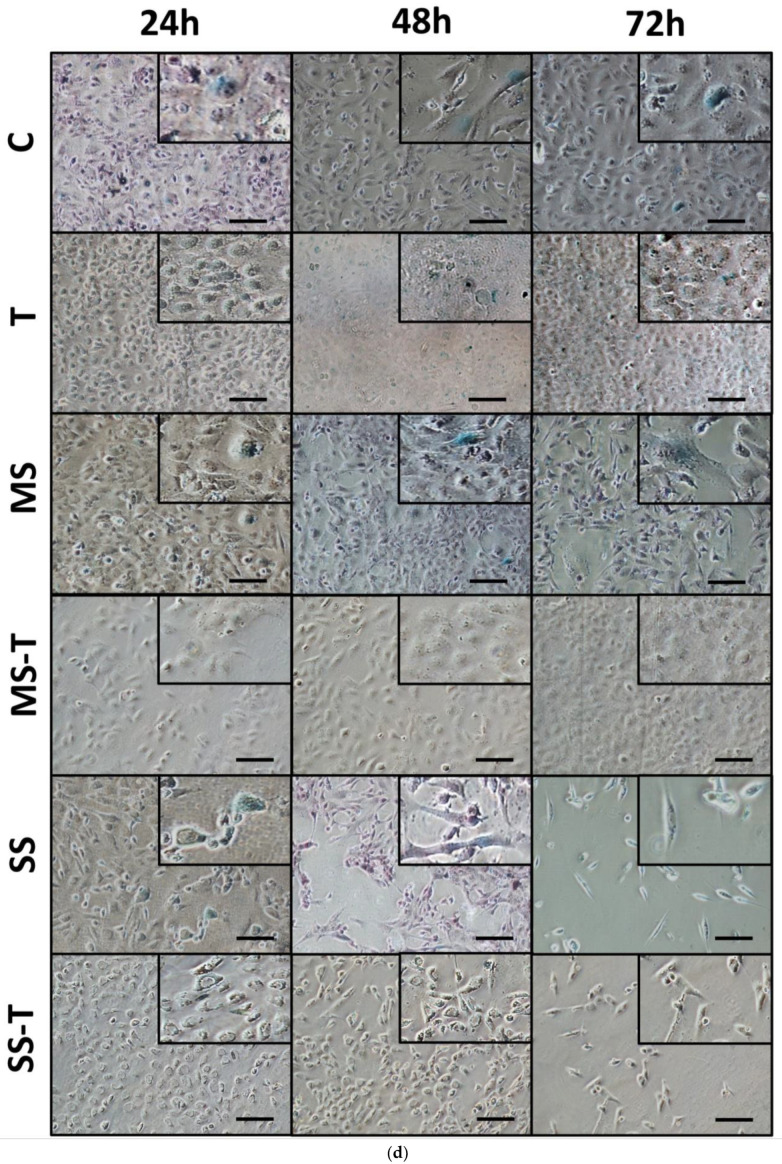
Senescence. HepG2 cells were cultured in control (C), mild steatosis (MS), or severe steatosis (SS) conditions. LX-2 cells were cultured in C, MS, and SS conditions. The addition of TGF-β rendered 3 further groups: T, MS-T, and SS-T. Senescence was assayed as the percentage of positivity for senescence-associated β-galactosidase (SA-βgal). (**a**) Representative micrographs of HepG2 cell senescence. (**b**) Percentage of senescence in HepG2 cells. (**c**) Percentage of senescence in LX-2 cells. (**d**) Representative micrographs of LX-2 HSC senescence. Data are shown as means ± SD and were analyzed using the Kruskal–Wallis test followed by Dunn´s post hoc test. Bars not sharing a letter are significantly different; *p* < 0.05. *n* = 6 wells from two independent experiments. Bars in micrographs = 100 µm.

HepG2 hepatocytes and LX-2 HSCs showed differential outcomes regarding senescence. On the one hand, MS and SS steatogenic conditions exerted opposite effects on senescence in HepG2 cells. MS increased the percentage of senescence compared with C and SS; in contrast, SS exhibited the lowest percentage of senescence at all the studied times compared with C and MS (Figure 4a,b).

On the other hand, senescence in LX-2 cells maintained a steady state during the incubation period under both C and T conditions. In MS, the senescence was low at 24 and 48 h, but it recovered to control levels by 72 h. Similarly, in MS-T, the senescence was low at 24 h; however, it recovered to control and T levels from 48 h. This suggests that TGF-β stimulation produces another cellular fate in MS, likely proliferation (as shown in Figure 2d). In SS, the senescence remained low regardless of the incubation time, probably due to increased cell death. SS-T only showed low senescence levels by 72 h, suggesting that HSCs exposed to high lipid contents are partially protected by TGF-β, but for a shorter time than HSCs under mild steatosis (Figure 4c,d).

## 4. Discussion

Steatotic liver disease is the most prevalent liver condition worldwide [1,2,16,17]; however, there is little evidence regarding the cellular effects of the steatogenic input level. In this study, we subjected two human hepatic cell lines (hepatocytes and HSCs, representing the major cell types involved in the development of the disease) to two levels of steatogenic conditions, mild and severe. Differential effects on these cells were observed depending on the steatogenic conditions as well as exposure time.

In HepG2 hepatocytes, steatosis was evident in both conditions, with an increased lipid content in SS compared with MS (Figure 1). Oleic and palmitic acids have previously been shown to be internalized by HepG2 cells [15,18,19,20]. A subtle change in the HepG2 cells’ morphology was observed in SS at 72 h, which led to a rounder shape compared with the previous classic polygonal shape (Figure 1a).

HepG2 cells showed sustained proliferation during the incubation time in control and steatogenic conditions. MS and SS exhibited lower rates of cell growth than C, and SS had the lowest. Moreover, the viability was lower in both MS and SS at 72 h. This slower cell growth in HepG2, with a smaller number of live cells, was associated with increased cell death. Therefore, we assessed apoptosis, since it is one of the main cell death pathways involved in chronic liver diseases, including steatotic liver disease [21,22]. Under the control conditions, the apoptosis rate was maintained at steady levels during the incubation period. MS significantly increased apoptosis at 72 h, whereas SS showed lower proportions of apoptotic cells, which discretely increased with the incubation time but did not reach the levels observed with MS or the control. This apoptosis behavior in hepatocytes under SS does not explain the low viability observed, suggesting that other death mechanisms might be involved. Other types of cell death besides apoptosis have been described, including pyroptosis and necroptosis [23]. Necroptosis has been shown to occur in the livers of patients with MASLD as well as in experimental models of the steatotic liver, such as the methionine–choline-deficient-diet-induced disease [24]. Pyroptosis has been described in chronic liver disease [25], and it possesses a closer relationship with inflammation due to the activation of the NLRP3 inflammasome complex [25,26,27]. Gaul et al. have suggested that NLRP3 released from hepatocytes can be engulfed by HSCs, inducing their activation and further exacerbating liver damage [26]. More research is needed regarding these death mechanisms in hepatocytes exposed to steatogenic milieus since they might help explain the pathophysiology of the progression from steatosis to steatohepatitis.

Senescence is another cellular mechanism involved in chronic liver disease, including steatotic liver disease [12,13,14]. During senescence, the cell cycle is arrested to avoid the proliferation of damaged cells, resulting in hepatoprotection against disease progression and carcinogenesis [28]. Our results in HepG2 showed a differential effect regarding the level of the steatogenic medium. On the one hand, MS increased the SA-β-gal activity; on the other hand, SS diminished senescence. These results suggest that a complex series of cellular mechanisms occur in hepatocytes during lipid overload, i.e., altering senescence, slowing proliferation, and increasing cell death (probably mediated by pyroptosis or necroptosis) as an attempt at hepatoprotection while also promoting disease progression.

LX-2 HSCs did not show Oil Red-stained lipid droplets as observed in HepG2. LX-2 cells were resistant to this staining, as shown previously, although Oil Red staining has been shown in primary cultured murine HSCs [29]. The control HSCs showed sustained cell growth during the incubation time. Slower cell growth was observed in T (TGF-β-stimulated) and MS HSCs, and even slower cell growth was observed in MS-T compared with the MS conditions. However, no cell growth was observed in SS or SS-T conditions. 

The HSCs’ viability was severely affected by exposure to fatty acids. MS diminished HSCs’ percentage viability by 72 h, suggesting that mild concentrations of fatty acids are tolerated for longer compared with severe conditions, where the viability progressively decreased over time. The TGF-β co-stimulation of HSCs in steatogenic conditions showed contrasting results related to the steatogenic input severity. In MS-T cells, TGF-β ensured cell survival at similar levels to the controls and TGF-β alone. The SS-T viability was significantly reduced from 24 h, dropping from 62% at 24 h to 6% at 72 h, suggesting that TGF-β stimulation cannot ensure survival in severe conditions. In the case of the HSCs, our data suggest that cell death was induced by apoptosis. Time was the determinant. HSCs in mild steatogenic input conditions exhibited more than 50% apoptotic cells in both MS and MS-T up to 72 h. In severe conditions, increased apoptosis was observed as early as 24 h, showing close to 50% in SS and 70% in SS-T, thus confirming the lability of HSCs in response to the level of steatogenic conditions. Senescence in HSCs was lower in both steatogenic conditions, which is consistent with the increased cell death observed. The HSC results might explain, at least in part, the lower fibrosis progression observed during steatosis. Activation is one of the most relevant issues when studying HSCs. We analyzed the percentage of α-smooth-muscle-actin-positive cells (Supplementary Figure S1), showing that TGF-β stimulation increased the percentage of positive cells compared with C. This was also observed for SS-T compared with SS. However, no differences were observed between MS and MS-T compared with the controls. This finding might be related to the insufficient amount of lipids to induce activation. More in-depth studies are needed to confirm or refute this observation. Previous reports have related HSC activation to the expression and activity of lipid-metabolizing enzymes [30,31]. Carnitine palmitoyltransferase, a rate-limiting enzyme in β-oxidation, was overexpressed in HSCs in both patients and murine models [31]. The pharmacologic inhibition of acetyl-coenzyme A carboxylase, which is involved in de novo lipogenesis, prevented HSC activation in a rodent model of nonalcoholic fatty liver disease [30]. This suggests that lipid challenge, fatty acid oxidation, de novo lipogenesis induction, and the presence of PNPLA3 polymorphisms involved in the steatotic liver might influence HSC activation and, thus, fibrosis progression [30,31,32].

To the best of our knowledge, this is the first report showing the outcomes of two levels of lipid overload in both hepatocytes and HSCs; however, more studies are needed to understand the effects of excess lipid content on these cells. The lipids used are also relevant. Using FFAs rather than neutral lipids, such as triglycerides, is expected to produce differential outcomes and hepatocellular damage. In this study, we used a lipid mix composed of two of the most common FFAs observed in hepatic triglycerides [33], palmitic acid, which is highly steatogenic and lipotoxic [34], and oleic acid, which is steatogenic and unsaturated and might act as an antioxidant [20]. FFAs are associated with increased cellular damage. In rats, increased diet-induced plasmatic levels of FFAs, but not triglycerides, were related to steatosis levels [35], while in mice, FFAs induced endoplasmic reticular stress and impaired autophagy [36].

This study’s main limitation is that the results only reflect the exposure of separate cells, hepatocytes and HSCs, to steatogenic conditions. A co-culture might provide more insight regarding their interaction during steatosis, as well as the possible protective role of hepatocytes over HSCs under lipid overload and the regulation of fibrogenesis.

Other cellular and molecular processes might be involved in the development and progression from steatosis to steatohepatitis, including pyroptosis, necroptosis, oxidative stress, and inflammation, which we did not cover in this paper.

## 5. Conclusions

Our results show that hepatocytes are more resilient under steatogenic conditions; however, they are still susceptible, showing decreased proliferation and lower viability with altered senescence. In contrast, HSCs are more sensitive to lipid overload, increasing cell death via apoptosis with decreased senescence and avoiding the proliferation of damaged cells. These differential effects should be related to the hepatocytes’ ability to metabolize and store fat and, consequently, protect other hepatic cells from lipotoxicity. HSC apoptosis might be related to the slow fibrogenic progression during steatosis and might help explain the progression from steatosis to steatohepatitis.

## Data Availability

Data are available at direct request.

## Supplementary Materials

The following supporting information can be downloaded at: https://www.mdpi.com/article/10.3390/cells13030282/s1, Figure S1: Activation of LX-2 cells. LX-2 cells were cultured in control (C), mild steatosis (MS), or severe steatosis (SS) conditions. The addition of TGF-β rendered 3 further groups: T, MS-T, and SS-T. Activation was assayed via flow cytometry as the fold of increase in expression of α-smooth muscle actin (α-SMA). (a) Dot plots of LX-2 cells for different conditions and incubation times. (b) α-SMA expression (fold change). Data are shown as means ± SD and were analyzed using one-way ANOVA followed by Tukey’s post hoc test. * *p* < 0.05 vs. C; ** *p* < 0.05 vs. SS. *n* = 3 wells for each condition.
