# Supplementary material for: In Vitro Lipid Overload Affects Cellular Proliferation, Apoptosis, and Senescence in a Time-Dependent Manner in HepG2 Hepatocytes and LX-2 Hepatic Stellate Cells
Source: Cells. 2024 Feb 4;13(3):282. doi: 10.3390/cells13030282 (PMC10854820; doi:10.3390/cells13030282)
Supplement: Supplementary file 1 [file cells-13-00282-s001.zip › cells-2794382-supplementary.pdf]

Supplementary Materials and Methods

Activation of LX-2 cells:

Alpha-Smooth muscle actine (α-SMA) was assessed by flow cytometry. An anti- α-SMA antibody coupled with phycoeritrine (PE, R&D systems, USA) was used. LX-2 cells were collected from C, T, MS, MS-T, SS, and SS-T conditions, washed with PBS, and fixed for 10 minuetes with paraformaldehyde 1% in cold PBS. After washing with PBS, cells were treated with 0.2% triton X-100 for 30 minutes at 4 °C. Cells were blocked with 1% bovine serum albumin in PBS for 15 minutes at room temperature. Ten µl of anti α-SMA-PE were added for every million cells, and incubated for 30 minutes protected from light. Cells were centriphuged and resuspended in staining buffer. The samples were read with a FACS Canto II (BD, USA) and analyzed using FACSDiva software. A total of 20 x 10^3 events were acquired in triplicate from 7.5 x 10^5 cells.

Supplementary Results

Increased activation of LX-2 cells was observed in T as an increased expression of α-SMA, this was also observed in SS-T compared with SS. However, MS-T did not show changes in α-SMA.

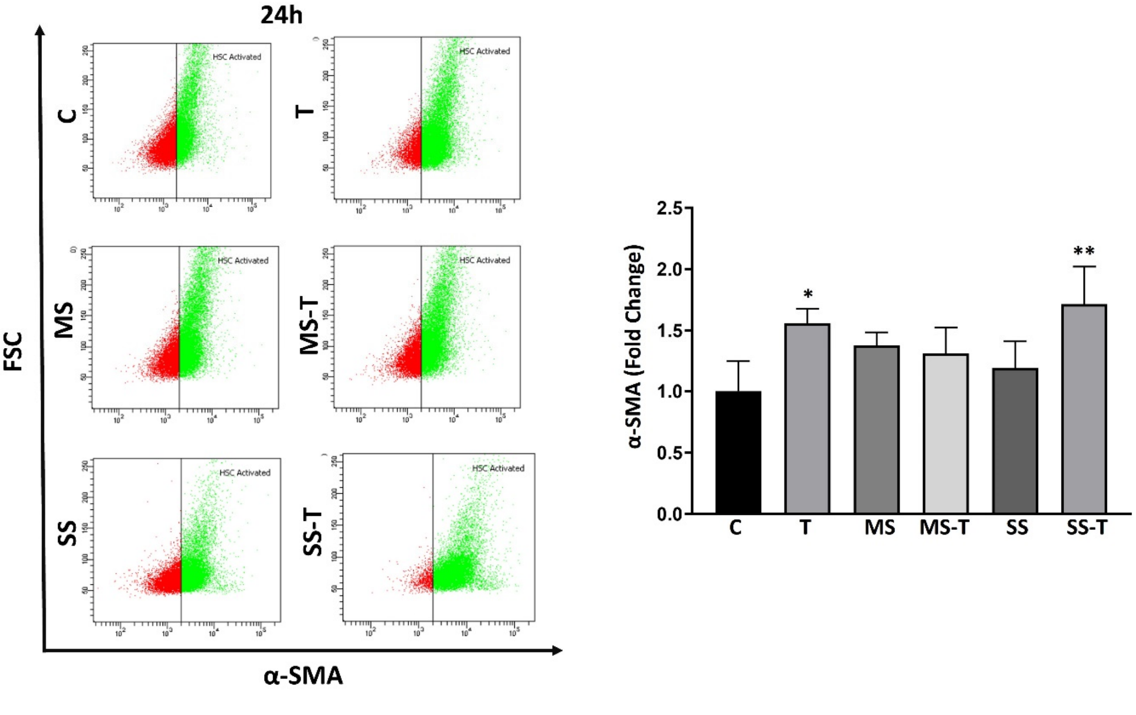

(**a**) (**b**)

Figure S1. Activation of LX-2 cells. LX-2 cells were cultured in control (C), mild steatosis (MS), or severe steatosis (SS) conditions. The addition of TGF-β rendered 3 further groups: T, MS-T, and SS-T. Activation was assayed via flow cytometry as the fold of increase in expression of α-smooth muscle actin (α-SMA). (a) Dot plots of LX-2 cells for different conditions and incubation times. (b) α-SMA expression (fold change). Data are shown as means ± SD and were analyzed using one-way ANOVA followed by Tukey's post hoc test. *$p < 0.05$ vs. C; **$p < 0.05$ vs. SS. n = 3 wells for each condition.
